# Nursing Students’ Perceptions of Spiritual Care in Türkiye: A Mixed Methods Study

**DOI:** 10.1007/s10943-025-02276-9

**Published:** 2025-02-17

**Authors:** Kamile Kırca, Emel Gülnar, Hüsna Özveren

**Affiliations:** Nursing Department, Faculty of Health Sciences, Kırıkkale University, Kırıkkale, Türkiye

**Keywords:** Nursing student, Spiritual care, Perception, Mixed method

## Abstract

Student nurses’ sensitivity and personal perceptions about spirituality and spiritual care are important in providing spiritual care because student nurses need to explore their spirituality and their relationship to care. The study was carried out with a sequential explanatory mixed method design. Quantitative data for the research was collected using the descriptive characteristics form and the spirituality and spiritual care rating scale. As a result of the data obtained from the focus group interviews, the authors identified five contexts, fifteen themes and many sub-themes. As a result of this study, it was determined that the student’s perceptions of spirituality and spiritual care were above average, and they saw spiritual care as a dimension of holistic care. However, they did not feel competent in providing spiritual care in clinical practice.

## Introduction

Spirituality and spiritual care have been important in health care and nursing education (Kannan & Gowri, 2020). Spirituality is a complex concept that is different for each individual. Spirituality varies according to an individual’s culture, development, life experiences, values, beliefs, and ideas about life (de Brito Sena et al., 2021). Spiritual care, on the other hand, is a patient-oriented, interpersonal, and integrative practice aimed at strengthening the spirituality of patients, increasing their commitment to life, making them at peace with their inner world, and eliminating their fears (Cruz et al., 2017; Sağkal Midilli et al., 2017). Most importantly, spiritual care focuses on the fact that humans are composed of matter and have a spiritual side (Bostancı Daştan & Buzlu, 2010).

### Nurses’ Role in Providing Spiritual Care

In the literature, there is evidence that in the absence of adequate moral support in the clinics, there are consequences associated with poor quality of life, dissatisfaction with nursing care, increased physical symptoms, moral distress, more aggressive treatment, and increased costs (Balboni et al., 2011; Harrad et al., 2019; Pearce et al., 2012; Rankin, 2018). Providing spiritual care or meeting the spiritual needs of patients is also defined by the World Health Organization as a core area of patient care, and particularly relevant guidelines and standards emphasize the importance of incorporating spiritual care into daily practice (Fitch & Bartlett, 2019).

In recent years, the number of studies on spirituality and spiritual care has been increasing rapidly, and the role of nurses in providing spiritual care has been emphasized (Aslan & Unsal, 2021; Cooper et al., 2013; Köktürk Dalcali & Erden Melikoğlu, 2022; Lewinson et al., 2015). Nurses are usually the first point of contact for spiritual concerns and needs in the hospital process. The essence of the nursing profession is to protect and maintain the integrity of the patient/healthy individual with all its dimensions. Spirituality is one of these dimensions. From this point of view, providing care regarding the spiritual dimension of the individual is also an important part of the nurse’s function (Çelik & Utas, 2016). To increase the quality of nursing care, the individual should be evaluated in all physical, psychological, social, and spiritual aspects and appropriate care should be planned. While giving this holistic care, the nurse is expected to include spiritual care in nursing practices (Ross et al., 2018).

Nurses, who provide continuous care to the sick individual, are expected to graduate by gaining knowledge, skills, perception, competence, and approaches to spiritual care starting from the student period. Student nurses’ sensitivity and personal perceptions about spirituality and spiritual care are important in providing spiritual care because student nurses need to explore their spirituality and their relationship to care.

### Spiritual Care in Nursing Education

Nursing students’ lack of knowledge and awareness of their spirituality affects their perceptive ability to provide spiritual care. The individual thinking system of the student nurse, her perspective on life, spiritual care, and her perception of spiritual care needs affects the quality of spiritual care (Dhamani, 2014). For this reason, it is very important for nursing students, who are candidates for the nursing profession, to recognize their values and analyze and control their reflections on care correctly. Clinical/educational nurses and academics should play an important role in keeping holistic care in the nursing focus and creating a good learning environment.

In nursing education, which is a journey from novice to expert, students need instructors’ guidance and role models. In addition, students need clinical experience with spiritual care to develop their understanding of diversity, reasoning, and knowledge about spiritual care. Students need to see and consider the assessment and application of spirituality in everyday nursing care as they do other nursing skills. Before graduating, students should learn to include spiritual care in the nursing process by diagnosing patients’ spiritual needs and habits, planning the nursing intervention for these needs, and implementing and evaluating the result (Giske, 2012). For this reason, the use of different and interactive educational methods such as group work, case discussion, role-playing, and cooperation with other disciplines are conducted.

Nursing education is about understanding patients’ spirituality. It will help students increase their awareness and develop their perception and skills. For this reason, this study was conducted to determine the spiritual care perceptions of nursing students who will be future healthcare professionals.

### The Contribution of Qualitative and Mixed Methods Studies

When the relevant literature is examined, it can be said that although there are similar studies in this field, the studies are not similar in terms of content, especially due to the differences in qualitative findings. The experiences reported by participants are varied, and this is attributed to the uniqueness of the individual. Qualitative research, through the direct words of participants, provides rich data to directly understand how they see and experience the world. The rich diversity of data from different groups of participants helps to make the research more inclusive and representative. Therefore, qualitative study results have a personal touch that cannot be achieved through other methods. This allows the results to be full of discoveries that were not foreseen at the beginning of the research, which makes the results unique. Mixed methods are ideal for addressing complex and multidimensional research questions. At the same time, spirituality and spiritual care are an integral part of holistic care for nursing students who are future health professionals.

Based on all these results, our study:

- An in-depth and multidimensional examination of nursing students’ perceptions of spiritual care using mixed methods will contribute to the literature.
- The qualitative analysis revealed new themes, concepts, and opinions about students’ perceptions of spiritual care.
- In addition, it is thought that this study may contribute to examining the effects and development areas of nursing students’ perceptions of spiritual care in the education process. It may provide suggestions for educational programs by evaluating the impact of nursing education on spiritual care.

## Methods

### Research Purpose

This study was conducted to determine the spiritual care perceptions of nursing students who will be health professionals in the future.

### Design

This study used a sequential explanatory mixed methods design comprising two phases (Schoonenboom & Johnson, 2017). Phase one entailed collecting quantitative data, with Phase Two collecting qualitative data to explain initial findings in greater depth. In Phase I, 257 nursing students were randomly selected to complete a survey regarding the spirituality and spiritual care rating scale. The results of Phase I provided valuable information to guide semi-structured interviews in Phase II. The researchers integrated qualitative and quantitative data and used qualitative themes to complement and expand the quantitative findings.

### Population and Sample

The study population consisted of 462 nursing students studying in the nursing department of a university in Türkiye. The study sample consisted of second, third, and fourth grade students who agreed to participate in the quantitative part (*n* = 257). The study excluded first-year students from the sample because they did not go to clinical practice. A total of 462 students enrolled in the university’s nursing department in the 2021–2022 academic year. One hundred and three of the students are first-year students. The study reached 72% of 359 students in the second, third, and fourth grades. Students did not take a course on spiritual care, but the subject of spiritual care is included in some courses such as palliative care.

The participants in the qualitative part of the research were determined by the “criterion-based strategy,” which is one of the sub-categories of the purposive sampling method, after analyzing the data obtained from the survey results. Criteria for collecting qualitative data: It was determined that the student nurses who participated in the research had “four and above scale score averages.” Since there is no definite rule such as interviewing a certain percentage of the whole sample in qualitative research (Erdoğan, 2014), the qualitative part of the research was terminated when data saturation regarding the research topic was reached (Yıldırım & Şimşek, 2013). For this purpose, three focus group interviews were conducted with 16 students who agreed to participate in the interview. Qualitative interviewing is a data collection technique that reveals participants’ perspectives and provides deeper and richer data on their experiences (Creswell & Plano Clark, 2018). The study conducted semi-structured interviews to determine nursing students’ perceptions of spiritual care.

The criteria for inclusion in the study were:

1. Being a student in the nursing department where the study was conducted
2. Having clinical experience to determine the students’ perceptions of spirituality and spiritual care to patients providing care (second, third, and fourth grades).
3. Being willing to participate in the study
4. Speaking Turkish
5. Being 18 years or older

### Data Collection Tools

In the quantitative phase of the research, data were collected using the descriptive characteristics form and the spirituality and spiritual care rating scale. The data were collected using a semi-structured interview form in the qualitative research phase.

### Descriptive Characteristics Form

It was prepared by researchers using literature (Abbasi et al., 2014; Boztilki & Ardıç, 2017; Chandramohan & Bhagwan, 2016; Harrad et al., 2019) to determine the introductory characteristics of nursing students.

### Spirituality and Spiritual Care Rating Scale

It is a five-point Likert-type scale with 17 items, developed by McSherry, Draper, and Kendrick in 2002, and a validity and reliability study was performed by Ergül and Bayık Temel in (2007) (Table 4). The rating of the articles is generated from one, meaning “totally disagree,” to five, meaning “totally agree.” The first 13 articles are graded inversely, and the last four are graded directly. As the total point average increases, the perception level of spirituality and spiritual care concepts also increases positively. As the total mean score increases, the perception of spirituality and spiritual care concepts also increases positively. A total scale mean score approaching 5 indicates a high level of perception of spirituality and spiritual care. If the Turkish version of the scale is used in our country, it is recommended that the sub-dimensions should not be evaluated separately. However, the scale should be evaluated on the overall score. The spirituality and spiritual care rating scale of the approximation of the total point average to five shows that the perception level of spirituality and spiritual concepts is high (Ergül &  Bayık Temel, 2007).

The scale consists of sub-dimensions of spirituality and spiritual care, religiosity, and individual care. In the study of McSherry, Draper, and Kendric, the Cronbach coefficient of the scale was 0.64 (McSherry et al., 2002). In our country, the validity and reliability tests of the scale were performed by Ergül and Bayık Temel, and Cronbach’s coefficient was found to be 0.76 within the scope of internal consistency. It has been suggested that the sub-dimensions should be evaluated over the overall score rather than separately if Turkish is used in our country (Ergül & Bayık Temel, 2007). In this study, the Cronbach Alpha coefficient of the scale was calculated as 0.85.

*Semi-structured Interview Form:* To determine the students’ views on their perceptions of spiritual care, non-directive, neutral, general nature, and open-ended questions were prepared to explore the process and its meaning. The questions in the interview guide were determined as a result of the researchers’ experiences and the relevant literature review (Cooper & Chang, 2022; Cordero et al., 2018; Kobya et al., 2019; Selman et al., 2018; Üzen Cura et al., 2022).

The semi-structured interview consists of open-ended questions about the participants’ views on spirituality and spiritual care. In addition, the prepared questions were taken from three different experts who have more than one study in the field of qualitative study, and their final form was formed by editing them.

The questions asked in the focus group interviews are as follows:

1. Please tell us your name and introduce yourself briefly.
2. What do you understand by the word “spirituality”?
3. What do you understand by “spiritual care”?
4. Do you think spiritual care should be provided in healthcare? If so, why? If not, why not?
5. What are the least helpful things a healthcare professional can do if a patient has spiritual concerns?
6. Is there anything we did not talk about that you think would be useful?

### Data Collection

Quantitative data of the research were collected through face-to-face interviews with individuals (*n* = 257) who participated in the research between March and April 2022 and filled in the scales by the individuals.

Qualitative research data were collected by conducting semi-structured interviews with 16 students in three focus groups between May and June 2022. Each focus interview consisted of five to six students. Since the high average score obtained from the spirituality and spiritual care rating scale indicates that their spiritual perceptions are high, interviews were conducted with students who had 4 points or more and volunteered to participate in the research.

In qualitative interviews, there is no definite rule about how many people the group will consist of and how many times. It is the researchers who will make this decision about what and how much they want to hear. However, some points should be considered in the negotiations.

Group size is significantly related to research questions, focus group type, and interview structure in focus group interviews. In this sense, the number of participants is also related to how well the researcher can keep the interview under control (Nyumba et al., 2018; Şahsuvaroğlu & Ekşi, 2013). According to Edmunds (1999), having more than ten people in the group may reduce the dynamics of the group, the interaction between the participants may lose its effect, and the control of the group may become difficult (Krueger & Casey, 2000; Nyumba et al., 2018). Each participant signed a written informed consent form before the interview. The interviews were conducted by a moderator and a reporter in quiet and well-lit classrooms where participants could freely express themselves.

The interview was conducted by sitting at the same level, listening actively, and being guided by the help of questionnaires. Researchers avoided judgmental and negative expressions and attitudes in the interviews. The answers given by the participants were stated exactly as stated in the findings section. Each interview lasted between 40 and 50 min. The interviews recorded with a voice recorder were transcribed. Researchers with previous qualitative research experience conducted interviews and analyses. COREQ-consolidated criteria for reporting qualitative research was used in the structuring and reporting phase of qualitative study.

### Data Analysis

The data obtained from the study were analyzed using the SPSS version 23.0 (statistical package for social sciences) software package. Number, percentage, mean, (min.–max.) and standard deviation values were used in the descriptive statistical evaluation of the data.

In the qualitative survey of Phase II, interviews were audio-recorded, and the recordings were transcribed for analysis. Colaizzi’s inductive qualitative content analysis method was used to analyze qualitative data. This approach consists of seven steps: (1) recording data, (2) selecting key phrases, (3) grouping themes, (4) defining and formulating meanings into themes, sub-theme clusters, and categories, (5) developing grouped themes and defining details, (6) expressing the researched phenomenon clearly, and (7) confirming whether the findings obtained overlap with their own experiences by interviewing the participants (Morrow et al., 2015). As a result, at the end of the analysis, themes and sub-themes were developed about the researcher’s and participants’ views on spiritual care. After the interview, a raw data document was created using audio recordings in Microsoft Word. Both researchers evaluated the data independently. A common consensus was reached after the themes were created. Researchers transformed the data into categories, themes, and sub-themes. During the analysis, a code was created for each participant, and their identities were thus kept confidential. In the research, the interviews were coded as N1, N2, N3… with the help of abbreviations to express the number of nursing students and participants. In the study, the interviews were coded as N1, N2, N3… with the help of abbreviations to express the number of nursing students and participants. Since the participants were student nurses, “Nursing” abbreviation “N” was used.

In general, the way of organizing the data, creating codes and themes, and transferring the data to tables and figures is followed (Creswell & Plano Clark 2018; Erdoğan, 2014). Content analysis is a type of analysis frequently used in qualitative research. The aim of content analysis is to reach facts and relationships between facts. The steps of content analysis consist of transcribing the data into text, first coding, second coding, creating themes and reporting (Erdoğan, 2014).

### Ethical Considerations

The study was approved by the Ethics Commission of the University (Date/number: 2021.12.10). Written permission was obtained from the university. All participants were informed about the purpose, procedure, and confidentiality of the study, and those who agreed to participate signed a voluntary informed consent form prior to participation. The study was conducted according to the ethical principles outlined by the World Medical Association’s Declaration of Helsinki.

### Results

The mean age of the students participating in the study was 21.15 ± 1.25, and 83.7% of them were female. 34.4% of students stated that they were studying in the third year, 85.2% of them heard about spiritual care, and 66.1% of them stated that they had received information about spiritual care before. 96.1% of students stated that it is necessary to provide spiritual care in the clinic, 88.7% did not provide spiritual care in the clinic, 94.9% of them stated that spiritual care is as necessary as physical care, 73.5% were self-sufficient in providing spiritual care, and 78.2% stated that they wanted to receive training on spiritual care (Table 1).

**Table 1:** Descriptive characteristics of nursing students (*N* = 257)

Characteristics	$\overline{x}$ ± SD	Min–Max
Age	21.15 ± 1.25	19–26
*n* %		
Gender		
Female	215	83.7
Male	42	16.3
Grades		
2nd	82	31.9
3nd	91	35.4
4nd	84	32.7
The state of hearing about spiritual care		
Yes	219	85.2
No	38	14.8
The state of getting information about spiritual care		
Yes	170	66.1
No	87	33.9
The situation of finding spiritual care in the clinic is necessary		
Yes	247	96.1
No	10	3.9
The state of giving spiritual care in the clinic		
Yes	29	11.3
No	228	88.7
The state of being self-sufficient in providing spiritual care		
Yes	68	26.5
No	189	73.5
The state of wanting to receive education on spiritual care		
Yes	201	78.2
No	56	21.8

SD, Standard deviation; Min–Max, Minimum–Maximum

The students’ spirituality perception sub-dimension religiosity mean score is 3.00 ± 0.61, individual care mean score is 2.86 ± 0.68, spirituality and spiritual care mean score is 2.79 ± 0.86, and spirituality perception scale mean score is 2.91 ± 0.41. (Table 2).

**Table 2:** Distribution of students’ mean scores on the spirituality perception scale and sub-dimensions (*N* = 257)

Scale	$\overline{x}$ ± SD	Min	Max
Spirituality	3.00 ± 0.61	1.00	4.50
Individual care	2.86 ± 0.68	1.50	4.75
Spirituality and spiritual care	2.79 ± 0.86	1.29	4.57
Total score	2.91 ± 0.41	1.94	4.00

SD, Standard deviation; Min–Max, Minimum–Maximum

### Qualitative Findings of the Study

The five contexts, themes, and sub-themes obtained in the research are given in Table 3.

**Table 3:** Context, themes, and sub-themes

Context	Themes	Sub-themes
Spiritual care components	Holistic care	Holistic approach
	Meaning and hope	To feel good
		Touch your heart
		Add meaning
		Joy of life
		A positive outlook on life
		Finding solutions to problems
		Reason for living
		Comfort
	Empathy	Empathize
		Be by your side
		Bear oneself well
		Love
		Smile
	Faith/Religion	Religion
		İnner balance
		Spiritual saturation
		Give spiritual care
Spiritual care need	Psychological	Making it valuable/ Valuing
		Provide trust
		Psychological treatment
	Communication	Need to be heard, to speak
		Good interpersonal relations
		To understand / to be understood
		Maintaining a social life
	Faith/Religion	Worship and prayer
		Kindliness
		To forgive, to be forgiven
Barriers to spiritual care	Working conditions	Workload redundancy
		Not having time / not wanting to spend time
	Devotion to the profession	Lack of passion for the profession
	Education	Lack of education
Spiritual care practices	Communication	Listen, explain
		Touch/meditation
		Smile
		Making a statement
	Fulfilling their religious beliefs	Fulfilling their religious needs
Spiritual care competence	Education	Training/seminars
		A spiritual care lesson should be added
	Working conditions	Workload should be reduced
		Must be a spiritual care nurse
	Providing a religious setting	Providing an environment to practice religious beliefs in clinics

### Spiritual Care Components

Students’ spiritual care was evaluated in terms of holistic care, meaning and hope, empathy, belief, and religiosity (Table 3). Students’ opinions on this subject:

*……. A little love, a little smile, and a nice word from the nurses, and caregivers increased his spirituality, peace of mind, and inner balance at that moment (N3).*

*…….. I think being nice to patients can be a part of spiritual care(N4).*

*……….We do not only provide physical care to the patient, we also need to be with him spiritually (N5).*

*…….. Spiritual care includes a holistic approach(N7)*

*.……..Spiritual care is care that requires empathy and understanding of people (N10).*

*……..…Providing care to the patient with a holistic approach, not only for the physical improvement of the individual but also psychologically (N2)*

### Spiritual Care Need

The students need spiritual care, psychological, communication, belief, and religiousness (Table 3). Students’ opinions on this subject:

*…… We must ensure that they adhere to the religious practices they have adopted (N9).*

*……… Smiling is also spiritual care. Making him feel valued (N7).*

*……… Spiritual needs, from a religious point of view, make us feel that we are next to him, making empathy, putting ourselves in his place, thinking about what he needs, and behaving accordingly with him (N16).*

### Barriers to Spiritual Care

Barriers to providing spiritual care were evaluated regarding working conditions, professional commitment, and education (Table 3). Students’ opinions on this subject:

*……. Working hours are too much, and they have too much workload, so they cannot spare time (N2).*

*….. Workload, nurses not receiving adequate spiritual care training (N5).*

*….. Their dislike for the profession may prevent them from doing spiritual care. (N10)*

### Spiritual Care Practices

Students learn spiritual care practices, communication, and fulfilling their religious beliefs (Table 3). Students’ opinions on this subject:

*…… Strengthening communication is spiritual care (N5).*

*…… It may provide the environment to fulfill their religious beliefs (N11).*

### Spiritual Care Competence

Applications to be made to give spiritual care to students were evaluated in terms of education, working conditions, and providing a religious environment (Table 3). Students’ opinions on this subject:

*….. Undergraduate education, communication courses and spiritual care courses in nursing can be added (N3).*

*…. Training and seminars can be organized (N11).*

*….. The number of nurses per patient should be increased, and working hours should be reduced (N6).*

*…… It would be great if a nurse could be assigned to each clinic under the name of spiritual care nursing (N16).*

## Discussion

Spiritual care is an important part of nursing (Giske, 2012). Nurses are in an ideal position to provide spiritual care and influence physical and mental health positively (Wu et al., 2012). In literature, mixed method studies investigating nursing students’ perceptions of spiritual care are limited (Cooper & Chang, 2022; O’Connell-Persaud & Isaacson, 2022; Pong, 2021).

Considering the complex and multidimensional nature of spirituality, the use of both qualitative and quantitative research designs, which will reflect the perspective of nursing students on spirituality and spiritual care, is the strength of our study and will contribute to literature. In this context, it was aimed to determine the spiritual care perceptions of nursing students and discussed in the light of the literature.

This study found that most nursing students believed that it was necessary to provide spiritual care in the clinic, heard about spiritual care, and were willing to receive more training on spiritual care. In addition, most of the students stated that they did not provide spiritual care during clinical practice and felt inadequate in this regard (Table 1). Kalkım et al. (2016) stated that 53% of nursing students did not find their knowledge of spiritual care sufficient, and 94.3% thought spiritual care was necessary. In the study of Aksoy and Çoban (2017), only 34.8% of the students stated that they gave spiritual care to their patients in clinical practice. In Cordero et al. (2018) study, the majority of nursing students (Portugal—87.1% and Brazil—73.7%) stated that they did not participate in an activity related to spirituality and health but were willing to do so.

In the same study, both groups of students stated that they should be prepared for religion and spirituality and that these subjects should be included in the curriculum (Cordero et al., 2018). These results show that although there are differences between countries, their views on nursing education are very similar. Similar to the studies, our study findings show that nursing students believe in the necessity of spiritual care, but they feel inadequate in providing care for these needs.

If nurses are not taught how to provide spiritual care meaningfully, they are unlikely to provide such care in practice, which can affect the quality of patient care. In Türkiye, as in the world, spiritual care is defined as a part of holistic care and is seen as multidimensional. However, it is stated that spiritual care receives little attention in nursing education in the world, and in our country, the nursing curriculum does not prepare nurses for spiritual care, spiritual activities are not clearly explained in the textbooks, and mentoring is not sufficiently mentioned (Daghan, 2018; Timmins et al., 2015; Pesut, 2008; Kalkım et al., 2018; Yilmaz & Gurler, 2014).

In recent years, with the increasing awareness of the healing power of spiritual care, studies to integrate spiritual care into nursing education are also continuing (Kalkım et al., 2018). It is important to include spiritual care and spiritual care practices in undergraduate nursing education, thus supporting nursing students in gaining spiritual care competencies before graduation. In this way, it is thought that students’ knowledge, attitudes, and awareness of spirituality and spiritual care will increase, and their ability to provide holistic care to patients will improve. To encourage adequate spiritual care in practice, it is necessary to integrate spiritual concepts, spiritual assessment tools, and spiritual care education into undergraduate nursing education.

In this study, it was determined that nursing students’ perceptions of spirituality and spiritual care were above average (Table 2). This demonstrated that students’ perceptions of spirituality and spiritual care are positive. When these positive perceptions of the students are supported by theoretical and practical training, they will be more willing to evaluate their spiritual needs and participate in spiritual care. In other words, the higher the spiritual care perceptions of student nurses, the higher the rate of including spiritual care in nursing practices (Chan, 2010). In addition, our study results are similar to those of the literature (Aslan & Unsal, 2021; Kalkım et al., 2016; Pour & Ozvurmaz, 2017; Üzen Cura et al., 2022).

In addition, it has been stated in some studies that the perception of spirituality and spiritual care in nursing students is not at the desired level (Gönenç et al., 2016; Kobya Bulut & Meral, 2019). It is thought that the differences between spiritual care perception scores in the literature may be due to both individual reasons and the fact that spirituality and spiritual care cannot find the same place in the nursing curriculum.

In this study, we determined that the students’ perceptions of spirituality and spiritual care were positive. The students received the highest score from the religiosity sub-dimension. This result may indicate that students attach importance to the religious dimension of spirituality. In addition, students scored above average in the individual care sub-dimension.

According to the qualitative study result of our study, students responded in a way that supports the quantitative finding by stating that religious beliefs should be fulfilled within the scope of spiritual care practices. Baysal and colleagues (2024), in their study with nurses working in Türkiye, Italy, and Albania, stated that nurses associate spiritual care with religion, and the reason for this result is that nurses living in these countries have religious beliefs. The study conducted by Chew and colleagues (2016) determined that the scores of nurses with religious beliefs were higher. In addition, these results can be reconciled with the religious and cultural characteristics of the society in which the participants live.

In this study, students’ expressions regarding spiritual care were grouped under four themes: meaning and hope, empathy, belief and religion, and psychology (Table 3). It is seen that the students defined spiritual care with many concepts. In addition, when the sub-themes are examined (finding solutions to problems, adding meaning, the joy of life, and the reason for clinging to life), students’ expressions can be found in the literature (Aslan & Unsal, 2021; Cordero et al., 2018; La Rocca-Pitts, 2015; Sherman & Free, 2019; Tiew & Drury, 2012) which is said to be similar to the expressions used to describe spirituality and spiritual care.

In addition, these findings suggest that students look at spirituality/spiritual care from a wide spectrum, not just from a religious perspective. In the study of Tiew and Drury (2012), it was stated that student nurses perceive spirituality as a universal and innate unifying force that enables individuals to be at peace and find meaning and purpose in life. In the study conducted by Aslan and Ünsal (2021), 50.7% of nursing students defined spiritual care as helping patients meet their spiritual needs, 44.5% as accepting and listening without prejudice, spending time with the patient, 43.6% showing empathy and compassion, 41% as referring to other professionals when they wanted to see a spiritual counselor (such as an imam) with a therapeutic touch, and 26.7% showing maintaining the family process.

In their study, Cordero et al. (2018) found that Brazilian nursing students have a more religious concept of spirituality, while Portuguese students think that spirituality has a more incredible combination of transcendent, intangible, ethical, and humanistic elements. These different concepts, drawn from different countries, highlight the difficulties of defining spirituality. Although spirituality has traditionally been associated with religious experiences, today, it is more concerned with deep cultural influences, understanding of life, and the pursuit of purpose, which may or may not arise from religion (Cordero et al., 2018).

To provide spiritual care, it is necessary to determine the spiritual needs of the individual. In our study, the students evaluated the spiritual needs of the patients within the scope of communication and belief/religious practices (Table 3). One of the students expressed this as “We must ensure that they practice the religion they adopt.” It makes us think that these statements of the students are mostly based on their observations during clinical practices; it draws attention to the need to strengthen the communication between patients, their relatives, and health professionals, and to help the patient meet his/her religious rituals (praying, reading religious books, meditating).

In our study, nursing students stated that working conditions, not being connected to the profession and lack of education, are the most important obstacles to providing spiritual care (Table 3). Although the students’ statements are similar to the literature (Green et al., 2020; Musa et al., 2019; Şahan & Terzioğlu, 2020), the statement of commitment to the profession draws attention. The fact that the nursing profession is preferred in our country due to its employment power makes us think that students are affected by this situation. On the other hand, reasons such as the high number of patients to be treated in the health system in our country and long working hours may have caused the students to state their working conditions as an obstacle.

According to the nursing students participating in the study, spiritual care practices were expressed as providing communication and fulfilling religious beliefs (Table 3). According to the nursing students who participated in the study, spiritual care practices were expressed as providing communication and fulfilling religious beliefs (Table 3). It is seen that these statements of the students are in line with their views on spiritual care needs. From here, it can be said that students can plan their spiritual care practices in line with the needs of the patients.

This situation brought to mind Govier’s (2000) definition of spiritual care: The nurse identifies the spiritual needs of the individual, meets and supports them with appropriate initiatives. However, it can be said that the spiritual care practices expressed by the students in the study are limited. In some studies, students’ spiritual care practices helping patients perform their religious rituals, praying for or with the patient, providing empathy, and psychological support, providing a comfortable environment, reading books, meditating, using therapeutic touch, singing, playing music for them, and finding alternative solutions to difficulties. It is stated that they perform many spiritual care interventions such as improving forgiveness, helping patients discover their expectations, and determining whether they are realistic (Connerton & Moe, 2018; Cordeo et al., 2018; Kalkım et al., 2016).

Students who are not good role models in practice do not see spiritual care as a duty and, therefore, do not give spiritual care to their patients in practice. It is necessary to apply educational methods such as learning scenarios, reflective thinking, case discussions, and patient simulation, especially during their academic education, to develop the spiritual caregiving aspect of students.

One of the important findings of our study was that the students felt inadequate about giving spiritual care (Table 3). The first question that comes to mind here is, what are the requirements for nurses to be competent in providing spiritual care? Spiritual care competence is defined as the knowledge, skills, and attitudes required to provide spiritual care (Fawcett, 2017; Ross et al., 2016). It was also stated that nurses’ perception of inadequacy was related to a lack of a clear understanding of what spirituality and spiritual care means and inadequate educational preparation (Cooper et al., 2013). Our study findings are also in parallel with literature.

Spiritual care for students has emphasized the necessity of creating an environment for religious practices in clinics, especially the lack of education, working conditions, and competence (Table 3). The importance of good collaboration between students, teachers, and clinical educators for optimal learning outcomes regarding spirituality and spiritual care has been noted in the literature (Giske & Cone, 2012). However, there is a need to learn more about this and develop guidelines on how best to facilitate this.

## Limitations

The limitation of this study is that the research was conducted in only one university in Türkiye. Therefore, in other institutions, the nursing students’ opinions could differ from what was found here.

## Conclusion

Quantitatively, the students’ perceptions of spirituality and spiritual care were found to be above average. It has been determined that most nursing students believe it is necessary to provide spiritual care in the clinic, but they feel inadequate about it. Qualitatively, it was seen that the students saw spiritual care as a dimension of holistic care and expressed their spiritual care needs, the obstacles encountered in providing spiritual care, spiritual care practices, and the requirements for spiritual care from a wide range of perspectives. It is recommended that further studies be conducted comparatively with a broader population and across various disciplines and universities. In nursing education, it is recommended to include teaching methods such as case discussions and patient simulation to increase students’ perception of spiritual care.

## Appendix 1

See Table 4.

**Table 4:** Spirituality and spiritual care rating scale

	Strongly disagree	Disagree	Uncertain	Agree	Strongly agree
I believe nurses can provide spiritual care by arranging a visit by the hospital Chaplain or the patient’s own religious leader if requested					
I believe nurses can provide spiritual care by showing kindness, concern and cheerfulness when giving care					
I believe spirituality is concerned with a need to forgive and a need to be forgiven					
I believe spirituality involves only going to Church/Place of Worship					
I believe spirituality is not concerned with a belief and faith in a God or Supreme being					
I believe spirituality is about finding meaning in the good and bad events of life					
I believe nurses can provide spiritual care by spending time with a patient giving support and reassurance especially in time of need					
I believe nurses can provide spiritual care by enabling a patient to find meaning and purpose in their illness					
I believe spirituality is about having a sense of hope in life					
I believe spirituality is to do with the way one conducts one’s life here and now					
I believe nurses can provide spiritual care by listening to and allowing patients’ time to discuss and explore their fears, anxieties and troubles					
I believe spirituality is a unifying force which enables one to be at peace with oneself and the world					
I believe spirituality does not include areas such as art, creativity and self-expression					
I believe nurses can provide spiritual care by having respect for privacy, dignity and religious and cultural beliefs of a patient					
I believe spirituality involves personal friendships, relationships					
I believe spirituality does not apply to Atheists or Agnostics					
I believe spirituality includes people’s morals					
